# Polyether Sulfone-Based Epoxy Toughening: From Micro- to Nano-Phase Separation via PES End-Chain Modification and Process Engineering

**DOI:** 10.3390/ma11101960

**Published:** 2018-10-12

**Authors:** Yann Rosetti, Pierre Alcouffe, Jean-Pierre Pascault, Jean-François Gérard, Frédéric Lortie

**Affiliations:** Ingénierie des Matériaux Polymères, CNRS UMR5223, INSA-Lyon, Université de Lyon, F-69621 Villeurbanne, France; yann.rosetti@insa-lyon.fr (Y.R.); alcouffe@univ-lyon1.fr (P.A.); jean-pierre.pascault@insa-lyon.fr (J.-P.P.); jean-francois.gerard@insa-lyon.fr (J.-F.G.)

**Keywords:** epoxy, polyether sulfone, epoxy toughening, polymer blends, microphase separation, nanophase separation

## Abstract

The toughness of a high-performance thermosetting epoxy network can be greatly improved by generating polyether sulfone−based macro- to nano-scale morphologies. Two polyethersulfones (PES) which only differ by their chain-end nature have been successively investigated as potential tougheners of a high-T_g_ thermoset matrix based on a mixture of trifunctional and difunctional aromatic epoxies and an aromatic diamine. For a given PES content, morphologies and toughness of the resulting matrices have been tuned by changing curing conditions and put into perspective with PES chain-end nature.

## 1. Introduction

Thermosetting (TS) polymers are materials of great interest in research and industry. Despite the growing importance of thermoplastic polymers, especially for their ability to be recycled, thermosets exhibit advantages that make them inescapable when high stiffness and strength or excellent thermal and chemical resistance are sought [1]. Particularly, epoxy prepolymers copolymerized with low molar mass diamines are suitable for composite applications since they own both a good processing ability and a high stiffness. However, highly crosslinked thermosets are also known to be very brittle. Thus, many efforts have been dedicated to improve the toughness of epoxy-based matrices by introducing reinforcing agents, such as functionalized rubbers, high-T_g_ thermoplastics (TP), core-shell particles [1,2,3,4,5] and more recently block copolymers (BCP) [6,7].

One of the most studied toughening strategies involves an initially soluble TP which subsequently suffers a Reaction Induced Phase Separation (RIPS) during the network formation leading to biphasic morphologies. This phenomenon is due to a loss of miscibility between the growing TS network and the TP, i.e., to the competition between reaction kinetics and thermodynamics of phase separation [2,3,4,8]. It has been proved that a biphasic morphology is required to get a significant improvement of the fracture toughness for a thermoset epoxy matrix [9,10,11,12,13,14,15].

Most of the related literature underlines the influence of the initial composition of the TS/TP blend (content in TP) on the resulting morphology and mechanical properties [4,5,11,12]. When TP content increases, the morphology typically goes from a dispersion of TP-rich nodules in TS matrix, to co-continuous phases and finally to phase inversion (TS-rich domains dispersed in a TP-rich matrix) [2,3,4,5]. However, with this kind of approach, two parameters vary at the same time: the content of TP and the morphology. As a consequence, it is difficult to isolate the sole influence of the morphology on the resulting properties.

A route to tailor the blend morphology by varying the polymerization conditions while keeping a unique initial composition was less explored [15,16,17,18]. Once gelation of the reactive TS system is reached, it is known that the morphology of a TS/TP blend is frozen and only phase purification can then take place until vitrification [2,3,4,5]. Thus, an easy way to tune TS/TP blend morphologies is to perform pre-curing at various temperatures (T_precure_) in order to take advantage of the competition between the occurrence of the TS gelation and the phase separation phenomenon. As a consequence, different morphologies could be developed starting from the same initial blend composition.

For the same initial blend composition, it is also possible to tailor the morphology of TP-rich separated phases by tuning the interactions between the TP chains and the TS prepolymers. Functionalizing chain-ends of the TP is a quite easy way to do so, since it modifies the miscibility between the TP and the TS in the initial state and along the TS polymerization. Kim and al. considered three polyethersulfones (PES) with different chain-ends groups mixed in an epoxy-amine system: a non-reactive one with chlorine and two reactive ones with hydroxyl or amine as chain-ends. Both later ones can react with oxirane groups from epoxy oligomers [8]. Finer morphologies were obtained with the PES having reactive chain-ends. The PES-rich separated domains have a diameter of about 10 to 100 nm range whereas micro-scale structures were obtained with the non-reactive PES. Unfortunately, the authors did not study the mechanical properties of the resulting TS/TP blends. On the opposite, Yoon and al. investigated the effect of the functionalization of a polysulfone in an epoxy-amine system which did not allow obtaining separated domains finer than 500 nm [12].

The scale (nano or micro) of the developed morphology is a very important parameter to consider managing a large toughening extent. It is now established that toughening of epoxies is more efficient when segregated domains are nanoscale rather than microscale since the TP particles/TS matrix interface area increases when the dimension of the dispersed phase decreases. In fact, as interactions exist between the two phases, a good adhesion between TP-rich phase and the thermoset matrix (continuous phase) contributes to the enhancement of toughness. In addition, according to the proposed mechanisms for toughened TS/TP blends [5], i.e., cavitation due to triaxial stress field ahead the crack front, shear yielding, crack bridging, and crack pinning, a larger number of particles dispersed at nanoscale will be much more efficient than fewer particles dispersed at microscale. Thus, many examples related to nano-segregated BCPs as tougheners of epoxy matrices were reported in the literature [6,7,19]. Basically one block of the copolymer must stay miscible in the matrix whatever the conversion while the other one has to phase-separate upon TS curing. It has also to be pointed out that most reported examples involve low glass transition temperature (T_g_) segregated blocks in order to favor contribution from a cavitation mechanism to the toughening. As a consequence, it can be concluded that the scale of the morphology developed in a TS/TP blend plays a key role in the final material properties. This aspect has not really been discussed so far, especially with high-T_g_ TPs as exemplified by the review on the TP-toughening of TS from Hodgkin et al. which only compare different types of morphologies without mentioning a possible dimension scale effect on reinforcement [14,20].

**Figure 1:**
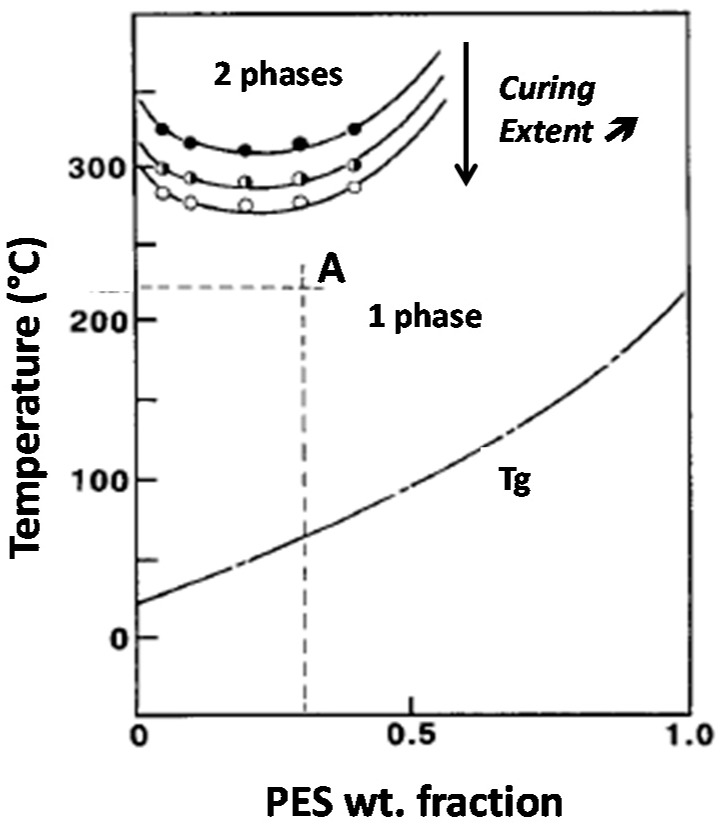
Phase diagram and initial vitrification curve of an epoxy-PES blend (LCST behavior). Critical solubility temperature decreases when curing extent increases. For instance, blend A is initially liquid and homogeneous will phase-separate upon curing.

This study put efforts in relating blend morphologies to the toughening efficiency of PES for a high T_g_ epoxy matrix. As it has been shown by Inoue and co-workers epoxy-PES blends exhibit a Lower Critical Solution Temperature (LCST) behavior, i.e., phase separation occurs if the mixture is heated above this critical temperature, miscibility being kept for lower temperatures [8]. The critical temperature for which phase-separation occurs and so the miscibility window decrease when the curing extent increases (Figure 1). This behavior is valid for binary epoxy-PES blends as well as for pseudo-binary blends when the amine hardener is also present. Such a phenomenon can be used to tailor morphologies by tuning curing conditions. Thus, starting from a given initial blend composition, two parameters will be considered in order to generate different types of morphologies: temperature of the first curing step, T_precure_ and PES chain-end nature.

## 2. Materials and Methods

### 2.1. Materials

**Figure 2:**
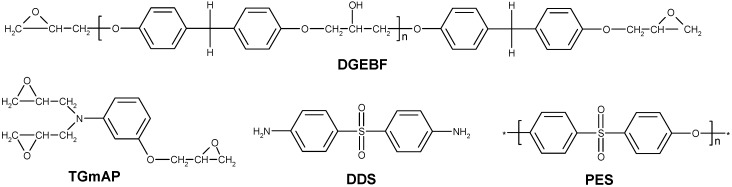
Structures of the involved chemicals.

A mixture of two epoxy prepolymers was chosen: the triglycidyl meta-amino phenol (TGmAP, Araldite MY0610, from Hunstman Co., Woodlands, TX, USA) and the bisphenol-F diglycidyl ether (DGEBF, Araldite GY281, from Hunstman Co., Woodlands, TX, USA) (Figure 2). Their epoxy equivalent weights were found to be 97.7 and 170.3 g·eq^−1^, respectively. Triglycidyl para-amino phenol (TGpAP) and DGEBA combinations are more often reported than these prepolymers in the literature. Nevertheless, TGmAP and DGEBF present some advantages: due to the meta-configuration on the trifunctional epoxy which leads to a higher modulus for a similar glass transition temperature (T_g_) than the para-configuration. The bisphenol-F based epoxy prepolymer is used since its viscosity is lower than the one of the bisphenol-A-based derivative.

The hardener was an aromatic diamine, the diamino diphenyl-sulfone (DDS, Aradur 9664-1, from Hunstman Co., Woodlands, TX, USA) (Figure 2). The diamine and the epoxy prepolymers were used as received, without further purification.

Two different polyethersulfones (PES) were used in this study (Figure 2). The first one is considered reactive since it is phenoxy-functionalized (reference 5003P, from Sumitomo Co., Hong Kong, China); the supplier indicates that it contains approximately a phenoxy group on half its chain-ends. The second one is a non-functionalized PES (reference 5200, from Sumitomo Co., Hong Kong, China). Both PES have similar molar masses since they present very close values of reduced viscosity (measured in DMF solutions), according to supplier information. The PES-5003P is reported to display an average molar mass of M_n_ ≈ 24,000 g·mol^−1^ [21,22]. PES-5003P will be referred as “f-PES” (for functionalized PES) and the PES-5200 as “nf-PES” (for non-functionalized PES).

### 2.2. Samples Preparation

The TGmAP/DGEBF weight ratio was taken equal to 80/20. The stoichiometry ratio, i.e., the initial amino hydrogen to oxirane functions ratio (r = a/e) was 0.9. An off-stoichiometry epoxy-amine with an epoxy excess is often used to limit the residual unreacted secondary amines [23].

Unmodified epoxy-amine matrices were prepared by dissolving DDS into the mixture of epoxy prepolymers at 80 °C in a glass reactor under mechanical stirring until homogeneity before curing.

The PES-modified epoxy systems required first a dissolution step overnight of the PES into the epoxy prepolymers at 130 °C under an inert gas. The weight percentage of PES was kept equal to 13% of the total weight of the PES-based systems. When total dissolution was achieved the system was cooled down to 80 °C and DDS was incorporated according to the same protocol than for the unmodified mixture. The PES epoxy-amine systems will be referred as “nf-PES” and “f-PES” systems.

For all the compositions, the amine dissolution step did not induce any reaction of the epoxy-amine system according to DSC analyses performed after dissolution procedure.

Before curing, the samples were degassed in a vacuum oven. The curing schedules were all composed of a first pre-curing step until the gelation of the systems (as discussed later), followed by a post-curing step at 180 °C for 2 h in order to get completion of epoxy-amine reactions.

### 2.3. Material Characterization

Viscosities were measured using an MCR-301 rheometer (Anton Paar, Graz, Austria) equipped with a disposable plate-plate geometry and a Peltier temperature controller. A fixed gap of 1 mm was chosen and measurements were carried out in permanent mode under a constant shear rate of 10 s^−1^.

Differential scanning calorimetry (DSC) measurements were conducted on a Q20 instrument (TA Instruments, New-Castle, DE, USA). For each analysis, about 3 to 5 mg of sample were placed in a hermetic pan capsule. Dynamic temperature ramps were made at a heating rate of 10 K·min^−1^ under nitrogen gas. Glass transition temperatures were determined at the onset point. Degrees of conversion were calculated from the enthalpy reaction of the exotherm peak (Equation (1)). T_0_ is the temperature at which the exothermic peak appears and ΔH_T_ is the total enthalpy of reaction.

(1) $$x=\frac{\int_{T_{0}}^{T}\frac{\mathrm{dH}}{\mathrm{dT}}\mathrm{dT}}{\Delta H_{T}}$$

Thermo-mechanical analyses of cured networks were performed using a Rheometrics Solids Analyzer RSA II (TA Instruments, New-Castle, DE, USA). The samples were tested in three-points bending conditions at a frequency of 1 Hz and a heating rate of 3 K·min^−1^.

Gelation times were measured using an ARES rheometer equipped with a disposable plate-plate geometry and a convective oven. A 1 mm gap was chosen. Gel times for isothermal curing were determined at the crossover of tanδ measured at different frequencies for a given temperature. Frequencies were selected from 1 Hz to 40 Hz and the angular strain was kept in the linear viscoelastic domain.

Scanning electron microscopy (SEM) analyses were conducted on fractured specimens used for mechanical testing. To reveal PES-rich domains, a chemical treatment using a potassium permanganate solution was applied on the sample surface before sputtering a 10-nm thickness Au-Pd coating [10]. An acceleration voltage of 10 kV was chosen for all pictures. Micrographs were recorded using a FEI Quanta 250 (Thermofisher Scientific, Hillsboro, OR, USA), except Figure 6B,C (see later in the text) recorded using a FEG S800 (Hitachi High Technologies, Tokyo, Japan) and Figure 11 (see later in the text) recorded with a FEI XL30 (Philips, Amsterdam, The Netherlands). FEG S800 has been replaced by FEI Quanta 250 during the course of the project. FEI XL30 is technologically equivalent to FEI Quanta 250 and has been used when the second one was not available.

Transmission electron microscopy (TEM) analyses were carried out using a CM 120 (Philips, Amsterdam, The Netherlands) under an acceleration voltage of 80 kV. Thin sections of about 70 nm were prepared by ultra-microtomy at room temperature.

The stress intensity factor, K_IC_, was measured on compact tension (CT) samples following the ASTM D5045 standard test recommendations [24]. The sample dimensions were 25 × 24 × 5 mm^3^. A loading rate of 10 mm·min^−1^ was chosen, the tests being carried out under controlled temperature (25 °C). Every given value is the result of a set of 10–15 samples.

In a first step, the samples were cut with a circular diamond-saw to realize a sharp notch. A razor blade was then used to initiate a natural crack. The K_IC_ values were calculated from Equation (2):

(2) $$K_{\mathrm{IC}}=\frac{F}{{\mathrm{BW}}^{\frac{1}{2}}}f(x)$$

where F is the force for the onset of crack growth, B the sample width, W the sample thickness, and f(x) a geometry factor calculated as following:

(3) $$f\left(x\right)=\frac{\left(2+x)(0.886+4.64x-{13.32x}^{2}+{14.72x}^{3}-{5.6x}^{4}\right)}{{\left(1-x\right)}^{3/2}}$$

x being the ratio between the initial length crack and the width of the sample.

## 3. Results

### 3.1. Definition of the Curing Schedules

**Figure 3:**
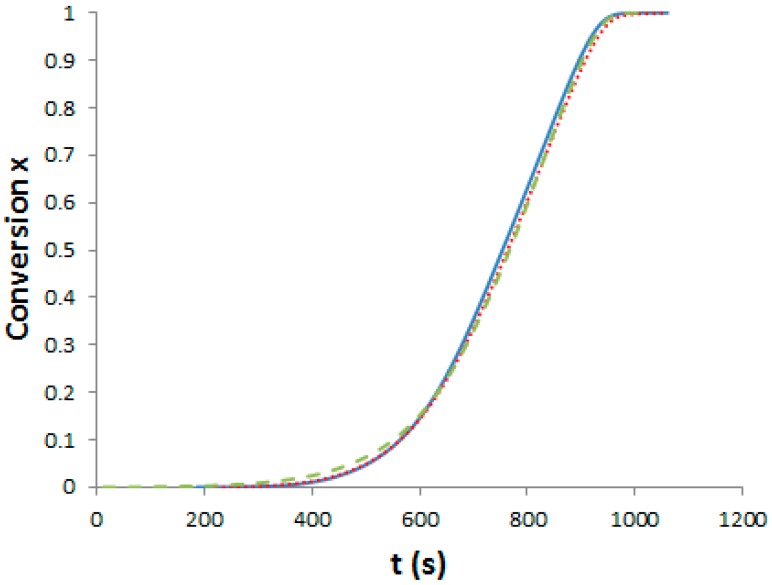
Evolution of the TS conversion versus time under non-isothermal conditions (10 °C/min). Full line: Neat system; dot line: f-PES system; dash line: nf-PES system.

As said, we need curing schedules composed of a 1st pre-curing step until the gelation of the systems, followed by a post-curing step at 180 °C for 2 h in order to get completion of epoxy-amine reactions. Our aim was to determine three different pre-curing steps for determining the effects on morphologies and properties. First, it is important to assess the influence of PES on polymerization kinetics. Evolution of the viscosity versus reaction time for the neat and the two modified epoxy systems under isothermal conditions, 150 °C has been measured (see Supporting Information, Figure S1). Dissolving the PES into the epoxy-amine system considerably increases its viscosity by more than one order of magnitude. It is noteworthy that PES is usually added as viscosity enhancing agent in semi-products like prepregs. To evaluate the effect of PES on the cure kinetics of the epoxy-amine network, epoxy conversion was measured by DSC for the neat and the two PES-modified systems. The systems were heated at 10 °C·min^−1^ from room temperature up to 300 °C. The exothermic peak corresponding to the heat of reaction appears above 100 °C and ends at about 280 °C (see Supporting Information, Figure S2). Assuming that a total conversion is reached at the end of the enthalpy peak, it is possible to plot the conversion of reaction as a function of temperature and subsequently as function of time (Figure 3).

Differences between the cure kinetics of the three systems are very slight. A dilution effect slowing down the initial reaction rate was observed for a TP-modified TS system [25] but in our case, the PES concentration is too low to induce this phenomenon. It is interesting to notice that the functionality of the phenoxy-end-modified PES does not lead to any change in the overall cure kinetics, even if –OH groups could have catalyzed the epoxy-amine reaction [1]. From these results, the same curing schedule can be considered for the neat and the PES-modified epoxy-amine systems.

Then the Conversion Temperature Transformation (CTT) phase diagram for the neat epoxy system has been determined [1]. As mentioned previously, gelation and/or vitrification determine the extent of phase separation and as a consequence final morphologies. It is therefore essential to determine at which conversion and time these physical transitions occur depending on the selected curing conditions.

The dependence of the glass temperature, T_g_, and the conversion, x, of a TS system could be fitted by the Di Benedetto model:

(4) $$\frac{T_{g}-T_{g0}}{T_{g\infty }-T_{g0}}=\frac{\lambda x}{1-\left(1-\lambda \right)x}$$

where T_g0_ and T_g∞_ are the glass transition temperatures of the non-reacted system and of the fully cured one, respectively. Pascault and Williams reported later that λ parameter corresponds to the ratio ΔC_p∞_/ΔC_p0_, with ΔC_p∞_ and ΔC_p0_ being the associated variations of isobaric calorific capacity from initial state to fully cured state, respectively [26].

**Figure 4:**
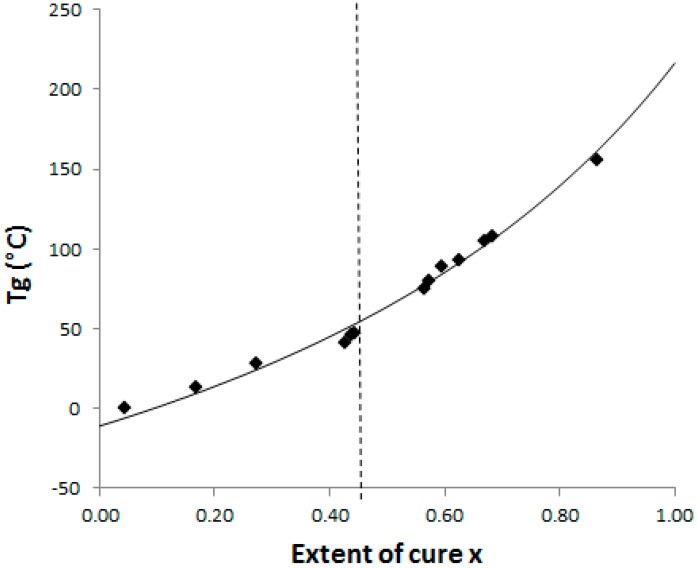
CTT phase diagram: dash line = gelation and full line = Di Benedetto curve glass transition temperature versus conversion for the neat epoxy-amine system; ◆: Experimental points.

The Di Benedetto dependence is plotted for the neat system (Figure 4) from experiments done in isothermal conditions at 130 °C. In such a complex (initially off-stoichiometric and excess of epoxy) reactive system, it is quite difficult to estimate when a total conversion is achieved. After 2 h at 180 °C, the system displays some residual heat of reaction, meaning that residual epoxies can react on themselves. The Di Benedetto dependence was then built thanks to a point corresponding to a not completely built epoxy network, as done by Jordan and al.: the point (x = 1; T_g∞_) is replaced by an intermediate (x_max_ < 1; T_gmax_) [27]. For the same reasons, it was impossible to experimentally determine a value of ΔC_p∞_. Thus the parameter λ was determined by fitting the Di Benedetto curve to the experimental points.

The values of T_g_ and ΔC_p_ for x = 0 (measured) and x = 1 (calculated) are listed in Table 1. Very close values for ΔC_p0_ were already found by Girard-Reydet and al. [28] and Eloundou and al. [29] (ΔC_p0_ = 0.54 J·g^−1^·K^−1^). Nevertheless, ΔC_p∞_ values differ (as the authors reported 0.19 J·g^−1^·K^−1^). Such discrepancies could be attributed to the stoichiometric ratio, r = 0.9 considered in this work, introducing some dangling chain ends.

To complete the CTT phase diagram, the conversion of the system at gelation, x_gel_ has to be known. It can be calculated thanks to the ‘Flory–Stockmayer’ equation [30]:

(5) $$x_{\mathrm{gel}}={\left[\frac{1}{\left(f_{e}-1\right)\left(f_{a}-1\right)r}\right]}^{1/2}$$

where f_e_ and f_a_ are the epoxy and amine functionalities respectively, r is the initial stoichiometric ratio. This calculation assumes that all the functions own the same reactivity and that no intermolecular reaction occurs. The nominal values of 2 and 3 were considered for the functionalities of the epoxy monomers (DGEBF and TGmAP respectively), and average epoxy functionality was calculated considering the mass distribution. Keeping in mind that the system is amine-deficient, x_gel_ was found equal to 0.45. This value was experimentally confirmed since the lower conversion for which insoluble species were evidenced in tetrahydrofurane (THF) was found to be 0.45 (complete solubility was observed for a conversion of 0.43). It will be considered that the conversion at gelation is not significantly affected by the presence of PES, as observed by Yu and al for anhydride-cured epoxy/PES blends with high PES contents (up to 20 wt %) if compared to our study [31].

As our aim is to know when phase changes (gelation and vitrification) occur during the curing schedules, it is now important to estimate the T_g_ value at the gel point, so called _gel_T_g_. With the help of this CTT phase diagram (Figure 4), _gel_T_g_ was found to be 55 °C. Based on _gel_T_g_ value and to ensure that the systems can reach gelation in a reasonable delay, the following curing temperatures have been considered: 90 °C, 130 °C and 150 °C. According to these temperatures, no vitrification effect will be suffered and morphologies will be definitively defined at gelation. Gel times have also been measured in these isothermal conditions by rheology under a multi-frequency mode using the tanδ cross-over (see Supporting Information, Figure S3) [29]. The apparent activation energy of gelation E_a_ could be determined since the gel time follows an Arrhenius’ law in respect to the temperature. A value of E_a_ = 64 kJ·mol^−1^ is found and is in agreement with results previously reported in the literature [32]. The gelation time and T_gx_ for the three different pre-curing temperatures are listed in Table 2.

For all the resulting networks, the glass transition temperature, T_gx_ is higher than _gel_T_g_, ensuring that gelation occurs during the pre-curing step. It could also be noted that for a pre-curing at 150 °C, vitrification is also reached but at longer times than gelation. These curing conditions will be applied both to neat epoxy-amine system and PES-based blends, followed by a post-curing at 180 °C during 2 h (see Supporting Information, Figure S4). It is then expected that these different curing schedules will bring important modifications in the developed morphologies for the PES-based systems.

### 3.2. Morphologies of the Epoxy/PES Blends

**Figure 5:**
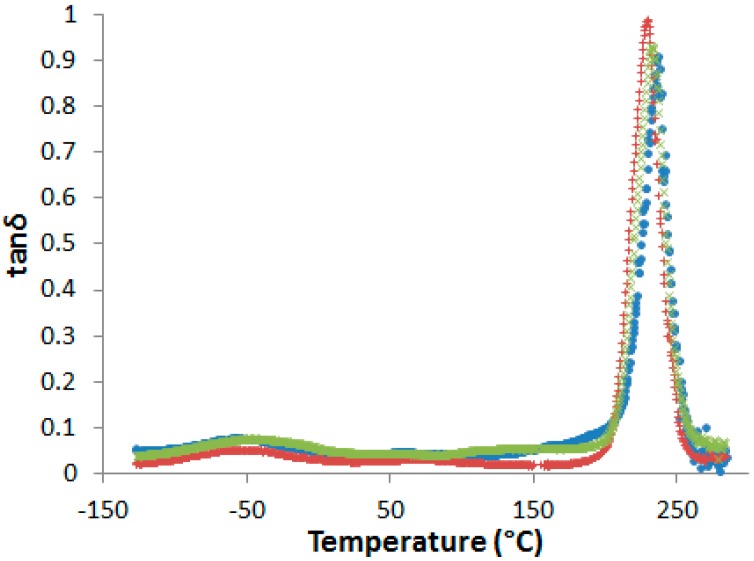
Dynamic mechanical spectra of the neat network and PES-based blends after pre-curing 1 h at 150 °C and post-curing 2 h at 180 °C (flexural mode, heating rate 3 K·min^−1^ at 1 Hz). •: neat network; ×: nf-PES blend; +: f-PES blend.

After curing, all the blends are transparent and brown-colored, i.e., the neat and the PES-modified epoxy-amine networks cannot be visually differentiated (see Supporting Information, Figure S5). No evidence of phase separation in cured samples could be evidenced by DSC or by dynamic mechanical analyses (DMA) (Figure 5).

In fact, even if a slight decrease of the α-peak position, T_α_, is observed in presence of PES, no other relaxation related to rich-PES separated phase can be detected on dynamic mechanical spectra. However, some authors observed a unique tanδ peak in phase-separated epoxy-PES blends due to the close values of the glass transition temperatures of the cured epoxy network and PES [33,34]. As a consequence, dynamic mechanical spectroscopy is not sufficient to state on phase separation.

**Figure 6:**
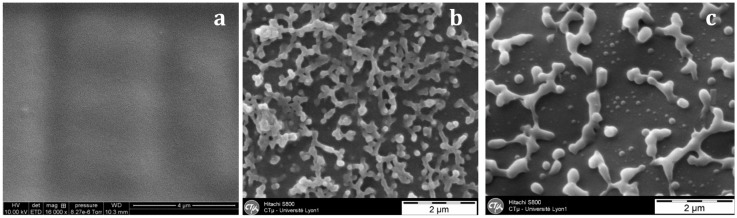
SEM observations on fracture surfaces (after a chemical treatment) of the nf-PES-based blends as a function of the curing temperature: 90 °C (**a**); 130 °C (**b**); 150 °C (**c**).

**Figure 7:**
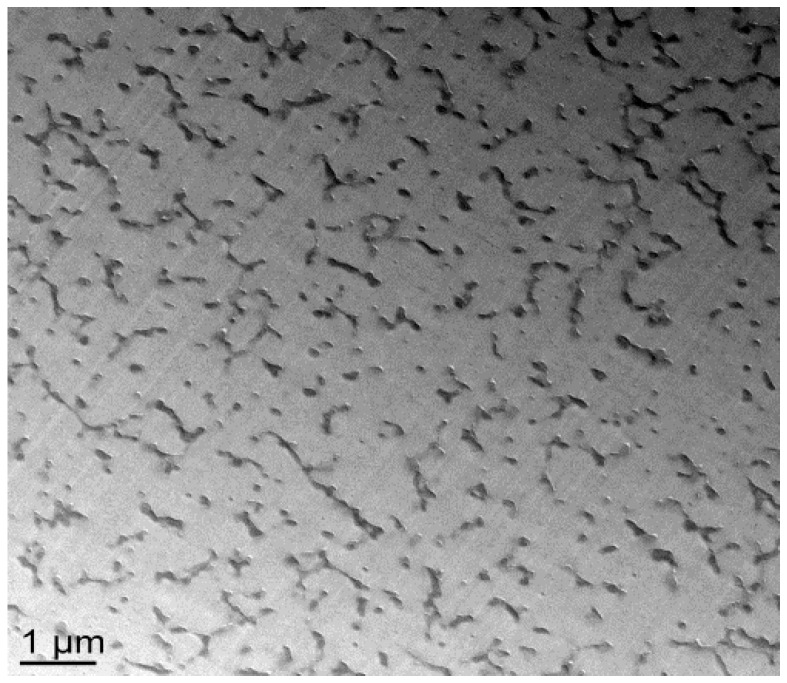
TEM observations of the nf-PES modified system cured at 150 °C.

With the nf-PES-based blend, the pre-curing temperature strongly influences the final morphology (Figure 6). By Scanning Electron Microscopy (SEM), it is not possible to evidence separated domains for blends cured at 90 °C. Besides, vermicular morphologies are observed for blends cured at 130 °C and 150 °C. For these vermicular morphologies, the average width of the PES-rich domains is about 250 nm for curing at 130 °C against 400 nm for curing at 150 °C. It has to be noticed that the samples were expected to be opaque or translucent and not transparent since the separated PES-rich domains having such dimensions use to scatter light. However, Inoue reported in a similar system that the PES and the epoxy/amine matrix own close refractive index, explaining why even microphase-separated materials can be transparent [20]. Transmission Electron Microscopy (TEM) clearly evidences the PES-rich phases (Figure 7). Furthermore, it can be seen that the vermicular morphologies developed in the nf-PES-based blends pre-cured at 130 °C and 150 °C do not lead to a PES-continuous phase, i.e., that PES nanostructures do not percolate. Vermicular morphology was also obtained by Akay and Cracknell for an epoxy network modified with a similar PES; they found continuity of the PES-rich phase at higher PES contents, i.e., 30 wt % [35].

For a better understanding of the mechanisms leading to a given morphology, it is important to know the value of the critical composition, Φ_TP,crit_, for phase-inversion. The ‘Flory-Huggins’ equation allows to estimate this composition [3,4].

(6) $$\frac{1}{\Phi_{\mathrm{TP},\mathrm{crit}}}=1+\sqrt{\frac{V_{\mathrm{TP}}}{V_{\mathrm{TS}}}}\frac{x_{w}(\mathrm{TP})}{\sqrt{x_{z}(\mathrm{TP})}}$$

Assuming a Schulz-Flory’ distribution of molar masses of the TP:

(7) $$x_{w}\left(\mathrm{TP}\right)=\frac{1+h}{1-h}$$

(8) $$x_{z}\left(\mathrm{TP}\right)=\frac{\left(1+4h+h^{2}\right)}{\left(1+h)(1-h\right)}$$

where V_TP_ and V_TS_ are the molar volumes of the thermoplastic polymer and the thermoset precursors respectively. h is the degree of polymerization of the TP.

This critical composition is calculated for the uncured epoxy-amine system. However, it has been showed that it does not suffer a significant change during curing of the TS system and as a consequence, it will be assumed to stay constant during all the curing process [36]. The phase diagram is then shifted only along the temperature axis during curing, due to the loss of miscibility between PES and the growing epoxy oligomers (Figure 1). Volumic fraction φ_TP,crit_ is found to be equal to 9%, i.e., when converted in weight fraction, φ_TP,crit_ = 11 wt %. Keeping in mind that PES has been initially introduced at ω_0_ = 13 wt %, it implies that the modified system is expected to go through a mechanism of spinodal decomposition [2,4,37].

As already mentioned, for nf-PES based blends, the higher T_cure_, the bigger the PES-rich separated domains are. As the phase separation proceeds via spinodal decomposition, co-continuous phases are formed in the early stages of the phase separation. The final morphology will depend on the competition between the thermodynamics of phase separation and the polymerization reaction rate. It has been shown that this competition usually leads to nodular morphologies for sufficiently long times before gelation, in order to decrease interfacial energy between phases [5,19]. In this study, the PES content is too low to induce co-continuous morphologies. The increase of the PES-rich phase size with T_precure_ can be explained in terms of molecular mobility when phase separation occurs. It can be argued that the conversion at which the phase separation takes place decreases when T_precure_ is increased since the LCST will be reached at a lower conversion (Figure 1). Thus the nodules which are formed in a less viscous matrix for high T_precure_ (since both conversion and viscosity are lower for higher T_precure_) have a higher tendency to coalesce and so get larger.

Another explanation concerning the vermicular structure could arise from the vitrification of the PES-separated phase during curing. In fact, after the phase separation, the two phases get purified, the matrix becoming richer and richer in epoxy-amine and as a consequence, the PES-rich separated domains richer and richer in PES. As a consequence, the glass transition temperature of the PES-rich domain increases since the PES has a much higher T_g_ than the epoxy-system before gelation [25]. It can be reasonably anticipated that the PES-rich phase vitrifies more easily for low T_precure_ temperatures, freezing the vermicular morphology. Unfortunately, no experimental evidence was found to validate this hypothesis even by using DSC. In fact, the glass transition of the PES-rich phase is always hidden by the residual enthalpy peak of the reaction of the remaining reactive species. Vitrification of the epoxy-rich matrix does not play a role on the morphology development as it occurs only for curing at 150 °C and a long time after gelation (Table 2). By the way, PES-rich phase owning a higher T_g_ than the epoxy-rich phase, would in all cases see its morphology fixed by its own vitrification. It is also known that the surface tension of an epoxy-amine system (prior to gelation) which is varying with conversion during curing and temperature can also interfere on the interfacial interactions between TS prepolymers and PES [38].

**Figure 8:**
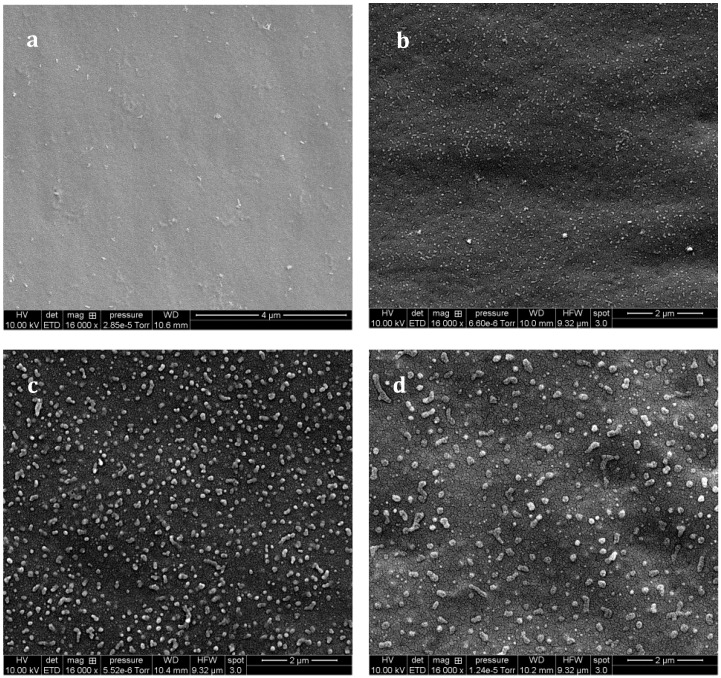
SEM observations (after chemical treatment) of the f-PES modified system as a function of T_cure_: 90 °C (**a**); 130 °C (**b**); 150 °C (**c**) and 180 °C (**d**).

The –OH end groups on the f-PES induce huge differences on the resulting morphologies of f-PES-based blends. As for the nf-PES, no phase separation could be evidenced by SEM for a blend cured at 90 °C (Figure 8a). For higher T_precure_, occurrence of phase separation is clearly evidenced, leading to nanometer-scale PES-rich domains. For curing temperatures from 130 °C to 180 °C, the higher T_precure_, the bigger the nodular domains: average diameter is about 40–50 nm for 130 °C (Figure 8b), and about 150 nm for 150 °C (Figure 8c) and even bigger than 200 nm for 180 °C (no pre-cure, Figure 8d).

Comparing resulting morphologies in the two PES-based blends, it can be seen that for both PES-based blends, the size of the dispersed domains increases with the pre-curing temperature, but more important is the fact that for a same T_precure_, the average size of the PES-separated domains is quite lower with f-PES than with nf-PES. As the two considered PES have quite similar molar masses, it can be considered that the two modified systems display the same φ_TP,crit_ value. Thus, such a huge difference between morphologies can only be explained by the presence of the phenoxy chain-ends of the f-PES. It is well known that phenoxy and oxirane are able to react to form an ether linkage [1,35]. Such a chemical reaction between f-PES and epoxy prepolymers can be expected in our system. Some phenoxy functions brought by the f-PES are then able to react with epoxy prepolymers to form an epoxy-PES block copolymer at the interface. For instance, Macosko and al. showed that compatibilizing a blend of two immiscible polymers inhibits the coalescence of the dispersed phase in the matrix, leading to quite finer morphologies [39]. Some authors also observed noticeable differences between non-reactive and reactive TP modifiers in a TS system. Yoon and al. managed to keep a NH_2_-terminated polysulfone modified epoxy-amine system homogeneous after curing whereas the non-reactive one leads to phase separation [12]. Finer morphologies were also obtained with the phase-separated reactive polysulfone if compared with the non-reactive one. A NH_2_-terminated PES was also successfully used by Kim and al. to greatly reduce the size of the separated domains of epoxy/PES blend compared with a non-reactive PES [8]. In this work, a similar phenomenon could result from the in-situ formation of an epoxy-PES block copolymer. One can wonder if the epoxy-PES copolymer formation takes place during the curing of the blends or if it already happened during the PES-dissolution step at 130 °C (See “Samples Preparation” part). Epoxy-PES mixtures obtained after PES-dissolution were analyzed via ^1^H Nuclear Magnetic Resonance (^1^H NMR) to evidence some differences between nf-PES and f-PES based blends. No concluding evidence was obtained due to the low amount of in-situ formed species.

Coming back to the behavior of the samples pre-cured at 90 °C. TEM analyses were carried out on these blends (see Supporting Information, Figure S6). Some PES-rich domains having an average diameter of about 20 nm are evidenced in both nf-PES and f-PES based blends, whereas a homogeneous structure is evidenced at nanoscale for the neat network. It proves that the PES-modified systems undergo phase-separation even when cured at 90 °C. It means that the two modified systems exhibit phase separation for all the curing temperatures considered in this study. Additionally, it can be stated that before gelation of the epoxy-amine system, the LCST is lower than 90 °C.

### 3.3. Fracture Properties 

It is now of great interest to investigate the effect of the developed morphologies exposed above on the matrix crack resistance of the PES modified systems. The possible toughening effects cannot be related to a PES-induced network plasticization (Figure 5). In fact, as reported before, only one relaxation peak associated to the glass transition of the epoxy-amine network is observed for the PES-based blends whereas it is established that the morphologies are biphasic. As two different α relaxation peaks corresponding to two phases in presence should be observed, it can be assumed that in our case the proximity of the T_α_ of PES and neat epoxy-amine network does not allow to clearly state on the existence of two phases.

Pure PES exhibiting a slightly higher T_α_ (239 °C) than the one of the neat epoxy-amine network (236 °C), it should then be expected that the T_α_ of PES-based blends is higher than the one of the neat epoxy-amine network. The lower T_α_ of the epoxy-amine matrix in the presence of PES could be related to a lower TS conversion. Taking into account the structures of the chemicals, it would not be surprising that the amine has more affinity than the epoxy prepolymers with the PES and is then retained longer in the PES-rich phase during purification even at high TS conversions: stoichiometry ratio would then be altered and the final properties modified. However, such a small difference in T_α_ after post-curing demonstrates that the epoxy-rich phase is only slightly modified, i.e., the intrinsic properties of the matrix such as crack resistance will not be different from neat epoxy-amine networks.

**Figure 9:**
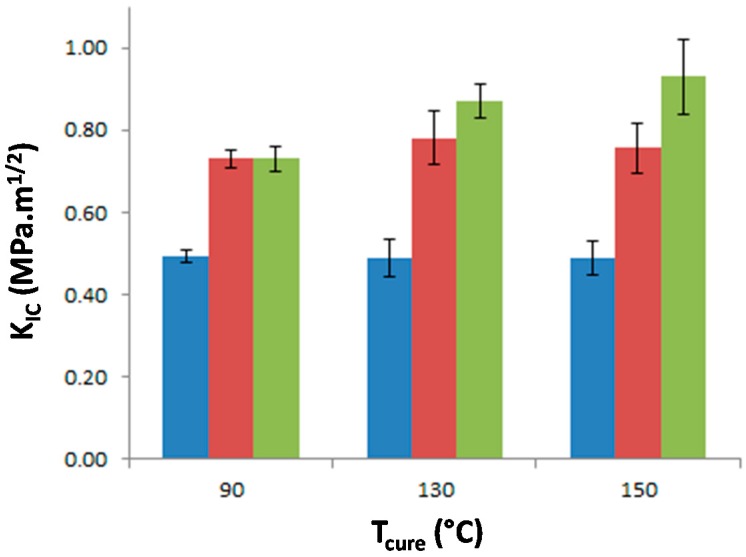
Stress intensity factor, K_IC_, of neat epoxy network and PES-based blends as a function of the curing temperature. Blue: neat epoxy; Red: f-PES based blend; Green: nf-PES based blend.

The stress intensity factor, K_IC_, of the materials was measured as a function of T_precure_ to investigate the effect of the morphology on the matrix crack propagation resistance (Figure 9). The neat epoxy-amine network appears very brittle as K_IC_ is equal to 0.49 MPa·m^1/2^ whatever T_precure_ which confirms that no significant effect on the network architecture is undergone. The incorporation of either f-PES or nf-PES dramatically modifies the fracture toughness for all T_precure_. Thus the PES-induced toughness improvement can solely be attributed to the generated morphologies since all epoxy networks exhibit similar architecture. The introduction of f-PES leads to an increase of K_IC_ up to 0.73 MPa·m^1/2^, i.e., 50% higher than for neat epoxy-amine network. For these f-PES-based blends, it is worth noting that the improvement in toughness does not depend on T_precure_ whereas the curing conditions have an impact on the final PES-rich domains size. In fact, considering spherical particles with an average diameter of 20 nm for the 90 °C-cured blend and 150 nm for the 150 °C-cured blend, the matrix-particles interface is about 50 times larger as T_precure_ is increased from 90 °C to 150 °C.

Very different results are obtained for blends based on nf-PES: the higher T_precure_, the higher the resulting K_IC_.

**Figure 10:**
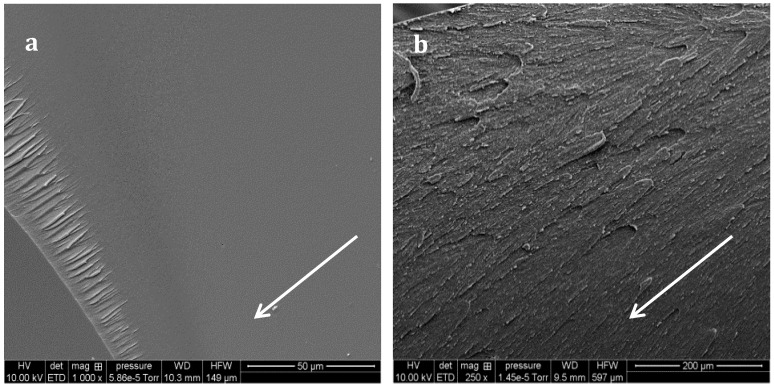
SEM observations of surface fracture for the neat (**a**) and f-PES modified (**b**) networks cured at 130 °C without any chemical treatment. Arrows indicate the propagation direction of cracks.

SEM micrographs of the non-chemically treated fractured samples allow identification of the toughening mechanisms. Neat epoxy-amine network presents a mirror-like surface (Figure 10a) whereas crack deviation is observed on PES-based blends (Figure 10b). Yee and al. proposed a list of potential TP-based toughening mechanisms, in particular for epoxies [5]. In our case the PES-separated domains are not ductile at room temperature since their glass transition temperature is close to 230 °C.

**Figure 11:**
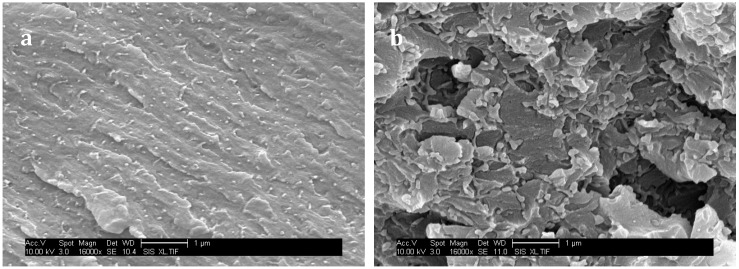
SEM observations of surface fracture for the f-PES (**a**) and nf-PES (**b**) modified networks cured at 150 °C without any chemical treatment.

Some toughening mechanisms are not possible in our systems: for instance TP particles bridging is only related to rubbery particles, and TP particles transformation implies semi-crystalline TP whereas PES is amorphous. Shear yielding could contribute to a large extent of the toughening effect. In fact, since epoxy-amine network and PS own different moduli (compression modulus for neat epoxy-amine network: 2.8 GPa, and PES: 1.6 GPa), stress concentration field around PES-rich domains superimpose which can induce yielding in a large volume of matter ahead from the crack tip. Kishi and al found that incorporation of PES in a DGEBA-DDS network allows a large increase of its ability to shear-yielding, evidenced by optical microscopic observations of compression samples under cross-polarized light [33]. An enhanced interfacial adhesion could have been expected with f-PES thanks to reaction between –OH chain-ends and epoxy-prepolymers [38]. However SEM micrographs of surface fracture of PES-based blends under a high magnification reveal that matrix/particle debonding occurred with both PES (Figure 11), i.e., interfacial adhesion is low. As a consequence crack pinning and bowing which both require high levels of interfacial adhesion are not expected to occur. Oppositely, multiple crack generation in the matrix, often observed in highly crosslinked matrices reinforced by rigid particles, should take place. Eventually, crack-path deflection is also one of the most probable toughening mechanisms.

For f-PES-based blends, the fact that toughness is independent of the PES-rich particle size could be explained by a “plateau effect”. In fact, for such nano-size morphologies, the particles are so numerous that their size does not influence any more the reinforcement extent. The two types of PES lead to a similar toughening effect for blends cured at 90 °C, which seems obvious according to the fact that average particles sizes are similar, as well as the matrix/PES interfacial adhesion. Higher values of K_IC_ obtained with vermicular morphologies in nf-PES-based blends can be explained by the fact that crack-path is much more altered than in the case of spherical-like domains-based morphologies. This phenomenon is highlighted by the large difference in roughness between the fractured surfaces (Figure 11a,b). With such elongated PES-rich domains, it can also be possible that a part of these domains are broken during crack propagation. PES being quite tougher (K_IC_ = 2.1 MPa·m^1/2^) than the epoxy-rich phase, toughening of the blend is again enhanced.

It is quite interesting to notice that these vermicular morphologies lead to a high toughening extent compared to other results on PES-reinforced epoxy networks reported in literature. In this study, an increase of 85% is obtained from neat epoxy network (K_IC_ = 0.49 MPa·m^1/2^) to nf-PES-based blends exhibiting vermicular morphologies (K_IC_ = 0.90 MPa·m^1/2^). To our knowledge, for similar PES contents, i.e., below 15–20 wt %, such an improvement of highly crosslinked epoxies was not already reported. For instance, Bejoy and al. managed to improve by 50% K_IC_ of DGEBA-DDS network thanks to a PES “P3500” from Gharda Chemicals [40]. Bucknall and Partridge also reached 50% improvement rate on networks based on tri and tetra-functional epoxy pre-polymers cured either by dicyanodiamide or DDS wih a PES “100P” from Victrex [41]. It is also surprising that some authors who worked with the same phenoxy-terminated PES used in this study (5003P from Sumitomo) did not observe any toughening enhancement on epoxy networks. For example, Kishi and al. considered DGEBA-DDS networks containing up to 15 wt % PES [34], as well as McKinnon and al. who worked on a triglycidyl para-amino phenol-DDS network which was not significantly toughen for PES contents ≤15 wt % [10]. Of course it has to be kept in mind that toughening improvement depends on several parameters which make direct comparison quite difficult: TP molar mass, curing schedule, final crosslinking density of the TS matrix, etc.

## 4. Conclusions

Several TS/TP morphologies have been prepared by reaction-induced phase separation of PES in a high performance thermosetting epoxy-amine network. For a constant PES content, morphologies were varied by either tuning the curing schedule or the nature of the PES chain-end nature. For this purpose, one non-functionalized PES (nf-PES) and one OH-end-functionalized PES (f-PES) which own the same molar mass were selected. Temperatures and durations of several pre-curing steps which must ensure TS gelation were first determined by plotting the CTT phase diagram. After checking that PES does not affect the TS curing kinetics, three pre-curing temperatures (T_precure_) have been chosen: 90 °C, 130 °C and 150 °C. Neat and modified matrices were then cured following a two-steps schedule: the isothermal pre-curing step followed by a post-curing step at 180 °C for 2 h.

A PES content (ω_0_ =13 wt %) close to the critical PES composition for phase-inversion was chosen. Therefore, the phase separation takes place following a spinodal decomposition mechanism. PES-modified systems exhibit a phase separation no matter what the chosen curing temperature is. In addition, the biphasic morphology varies with the curing conditions and the chain-end nature of the PES. For both PES-modified systems, the higher T_precure_ is, the larger the PES-rich domains are.

- For T_precure_ = 130 °C and 150 °C, separated PES-rich domains are large enough to be observed by SEM. The non-functionalized PES leads to vermicular morphologies with typical width of some hundreds of nanometers; whereas the –OH functionalized PES leads to nodular morphologies of finer dimensions (some tenths of nanometers).
- For T_precure_ = 90 °C, very fine PES-rich domains are obtained and were only observed by TEM which offers a better resolution than SEM. The dimension of these domains does not permit to highlight any difference in shape between the two PES-based blends morphologies.

The fact that the OH-bearing PES leads to finer morphologies is explained by the surfactant effect of the PES-epoxy block copolymers, assumed to be formed in situ since oxirane and phenoxy groups are able to react together.

The modified systems significantly exhibit higher toughness (from 0.73 MPa·m^1/2^ to 0.90 MPa·m^1/2^) than the neat one (0.49 MPa·m^1/2^). The major toughening mechanism is supposed to be crack-path deflection while micro-cracking and shear-banding of the epoxy-rich matrix may also be envisioned. For vermicular morphologies, the crack may also go through a part of PES-rich domains which are intrinsically tougher than the epoxy-rich matrix, leading then to a better toughening if compared to nodular morphologies.

For further possible industrial applications, f-PES leads to the same toughening extent no matter the selected curing schedule, which is important in terms of reliability and repeatability. Besides, nf-PES toughening extent depends on the chosen curing schedule; fortunately, the better properties are obtained for high T_precure_ which imply shorter processing cycles (but it may also bring higher risks of exothermicity issues in the case of massive parts manufacturing).

## Figures and Tables

**Table 1:** Glass transition and isobaric calorific capacity values for the neat epoxy-amine system at x = 0 and x = 1.

T_g0_ (°C)	T_g∞_ (°C)	ΔC_p0_ (J·g^−1^·K^−1^)	ΔC_p∞_ (J·g^−1^·K^−1^)
−10.8	216	0.53	0.27

**Table 2:** Glass transition temperature and conversion of the neat system as a function of the cure temperature and duration, x values (at the end of each pre-curing step) are evaluated from the Di Benedetto curve of the CTT phase diagram.

T_cure_ (°C)	T_cure_ (h)	T_gx_ (°C)	x
90	24	75	0.55
130	3	122	0.74
150	1	156	0.85

## Supplementary Materials

The following are available online at http://www.mdpi.com/1996-1944/11/10/1960/s1, Figure S1: Evolution of the viscosity versus time for epoxy-amine-based systems cured under isothermal conditions. Full line: neat system; dotline: f-PES system; dash line: nf-PES system, Figure S2: Enthalpy of reaction measured by DSC under dynamic curing conditions (10 °C/min); Blue: neat epoxy-amine system; Red: f-PES system; Green: nf-PES system, Figure S3: Variation of tan δ with reaction time for various frequencies (from 1 Hz to 40 Hz) during isothermal curing at 150 °C for the neat epoxy-amine system, Figure S4: Scheme representation of the curing schedules considered in this study, Figure S5: Visual aspect of the neat epoxy-amine network (a); the f-PES-based network (b) and the nf-PES-based network (c), Figure S6: TEM micrographs of the neat epoxy-amine network (a); f-PES-based blend (b) and nf-PES-based blend (c) cured at 90 °C.
