# Supplementary material for: Polyether Sulfone-Based Epoxy Toughening: From Micro- to Nano-Phase Separation via PES End-Chain Modification and Process Engineering
Source: Materials (Basel). 2018 Oct 12;11(10):1960. doi: 10.3390/ma11101960 (PMC6213164; doi:10.3390/ma11101960)
Supplement: Supplementary file 1 [file materials-11-01960-s001.pdf]

materials

MDPI

Supporting Information:

Polyether sulfone-based epoxy toughening: from micro- to nano-phase separation via PES end-chain modification and process engineering

Yann Rosetti, Pierre Alcouffe, Jean-Pierre Pascault, Jean-François Gérard and Frédéric Lortie *

Ingénierie des Matériaux Polymères, CNRS UMR5223, INSA-Lyon, Université de Lyon, F-69621 Villeurbanne, France; yann.rosetti@insa-lyon.fr (Y.R.); alcouffe@univ-lyon1.fr (P.A.); jean-pierre.pascault@insa-lyon.fr (J.-P.P.); jean-francois.gerard@insa-lyon.fr (J.-F.G.)

* Correspondence: frederic.lortie@insa-lyon.fr; Tel.: +33-4-7243-6124

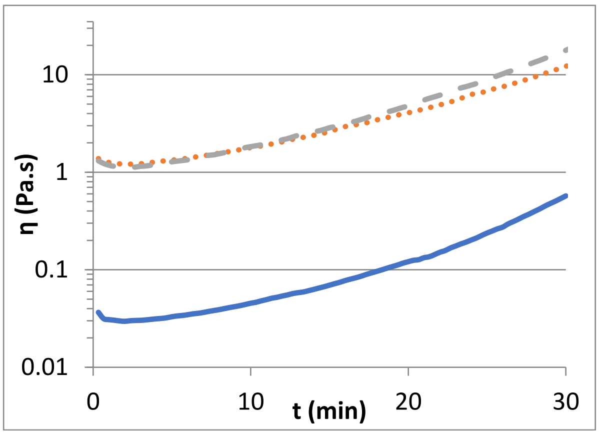

Figure S1. Evolution of the viscosity versus time for epoxy-amine-based systems cured under isothermal conditions. Full line: Neat system; dot line: f-PES system; dash line: nf-PES system.

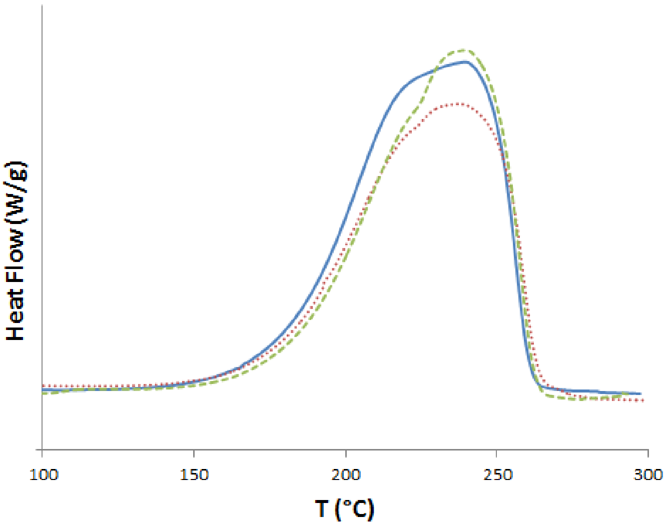

Figure S2. Enthalpy of reaction measured by DSC under dynamic curing conditions (10 °C/min) Blue: neat epoxy-amine system; Red: f-PES system; Green: nf-PES system.

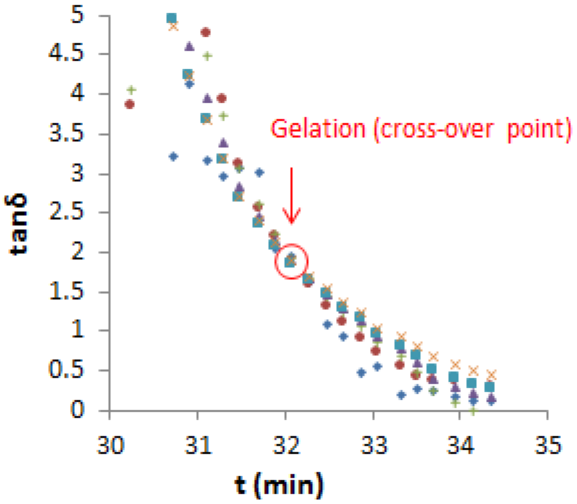

Figure S3. Variation of tan δ with reaction time for various frequencies (from 1Hz to 40 Hz) during isothermal curing at 150 °C for the neat epoxy-amine system.

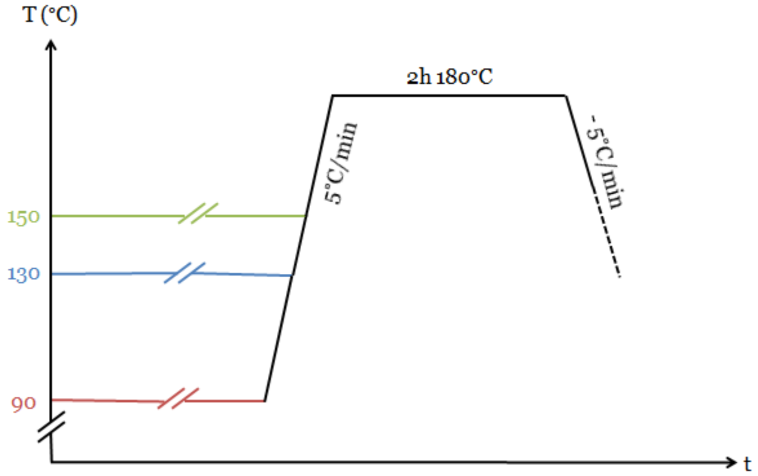

Figure S4. Scheme representation of the curing schedules considered in this study.

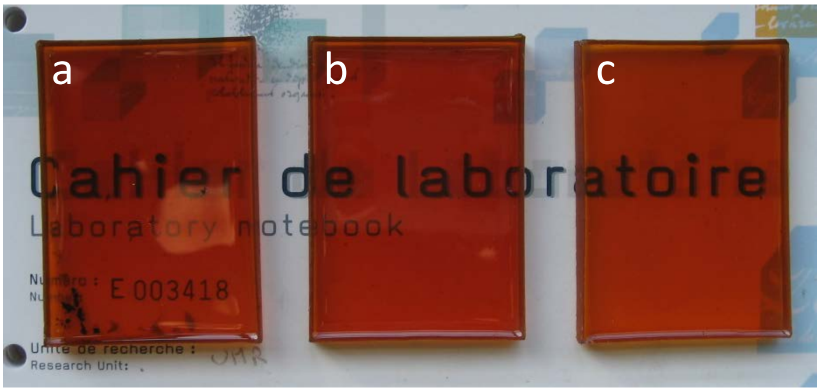

Figure S5. Visual aspect of the neat epoxy-amine network (**a**); the f-PES-based network (**b**) and the nf-PES-based network (**c**).

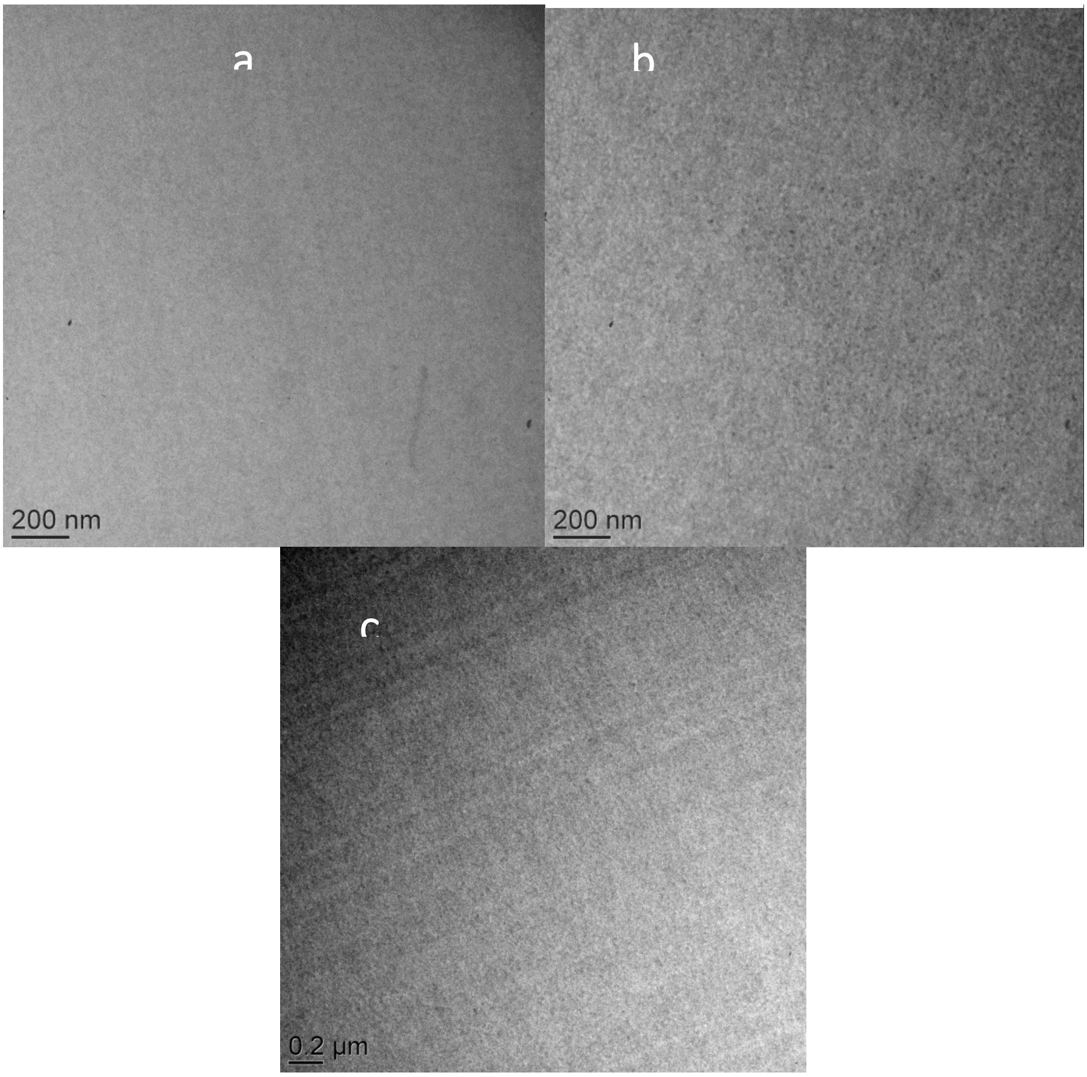

Figure S6. TEM micrographs of the neat epoxy-amine network (**a**); f-PES-based blend (**b**) and nf-PES-based blend (**c**) cured at 90 °C.

© 2018 by the authors. Submitted for possible open access publication under the terms and conditions of the Creative Commons Attribution (CC BY) license (http://creativecommons.org/licenses/by/4.0/).
